# Learning anatomy changes from patient populations to create artificial CT images for voxel‐level validation of deformable image registration

**DOI:** 10.1120/jacmp.v17i1.5888

**Published:** 2016-01-08

**Authors:** Z. Henry Yu, Rajat Kudchadker, Lei Dong, Yongbin Zhang, Laurence E. Court, Firas Mourtada, Adam Yock, Susan L. Tucker, Jinzhong Yang

**Affiliations:** ^1^ Departments of Radiation Physics The University of Texas MD Anderson Cancer Center Houston TX; ^2^ Bioinformatics & Computational Biology The University of Texas MD Anderson Cancer Center Houston TX; ^3^ Scripps Proton Therapy Center San Diego CA; ^4^ Department of Radiation Oncology Christiana Health Care Systems Newark DE USA

**Keywords:** active shape models, principal component analysis, deformable image registration, validation

## Abstract

The purpose of this study was to develop an approach to generate artificial computed tomography (CT) images with known deformation by learning the anatomy changes in a patient population for voxel‐level validation of deformable image registration. Using a dataset of CT images representing anatomy changes during the course of radiation therapy, we selected a reference image and registered the remaining images to it, either directly or indirectly, using deformable registration. The resulting deformation vector fields (DVFs) represented the anatomy variations in that patient population. The mean deformation, computed from the DVFs, and the most prominent variations, which were captured using principal component analysis (PCA), composed an active shape model that could generate random known deformations with realistic anatomy changes based on those learned from the patient population. This approach was applied to a set of 12 head and neck patients who received intensity‐modulated radiation therapy for validation. Artificial planning CT and daily CT images were generated to simulate a patient with known anatomy changes over the course of treatment and used to validate the deformable image registration between them. These artificial CT images potentially simulated the actual patients' anatomies and also showed realistic anatomy changes between different daily CT images. They were used to successfully validate deformable image registration applied to intrapatient deformation.

PACS number: 87.57.nj

## I. INTRODUCTION

With recent progress in the development of highly conformal radiotherapy techniques, such as intensity‐modulated radiotherapy (IMRT), volumetric‐modulated arc therapy (VMAT), and proton therapy, deformable image registration (DIR) has become very important to radiation therapy in compensating for nonrigid variations, automatically delineating target volumes and normal tissue contours, and determining dose accumulation for plan evaluation.^1^, ^2^, ^3^, ^4^, ^5^, ^6^ This trend has been clearly demonstrated in recent years as more and more FDA‐approved commercial DIR tools have become available in clinical radiation therapy, such as MIM Maestro (MIM Software, Cleveland, OH), VelocityAI (Velocity Medical Solutions, Atlas, GA), SPICE in Pinnacle^3^ treatment planning system (Philips Healthcare, Cleveland, OH), Smart Segmentation in Eclipse treatment planning system (Varian Medical Systems, Palo Alto, CA), and atlas‐based segmentation in RayStation treatment planning system (RaySearch Laboratories AB, Stockholm, Sweden).

However, the validation of DIR algorithms, particularly in terms of accuracy, has long been very difficult,^7^, ^8^, ^9^, ^10^, ^11^ mainly owing to the lack of the ground truth of the deformation. A concerted effort has been made to validate DIR algorithms for radiation therapy.^12^, ^13^, ^14^, ^15^, ^16^, ^17^, ^18^, ^19^, ^20^, ^21^ Currently, the most common validation method is based on physician‐drawn structure contours or physician‐picked anatomical landmarks.^12^, ^15^, ^18^ This validation method is generally time‐consuming and labor‐intensive when many contours or landmarks needed to be delineated or picked manually, and the method inevitably suffers from inter‐ and intraobserver variability. In addition, this validation method is limited because it cannot provide voxel‐by‐voxel validation, which is particularly important when the DIR is applied to dose accumulation during treatment planning.

Another DIR validation method uses anthropomorphic phantoms that can be physically deformed by a known amount. For example, Kirby et al.^19^, ^20^ built a head and neck phantom and a pelvic phantom that contain glow‐in‐the‐dark optical markers that are CT transparent so the optical markers do not influence DIR results on CT images. When the phantoms are deformed, the deformation vector field (DVF) can be measured optically and compared with the calculated DVF of DIR algorithms. However, these phantoms cannot mimic realistic situations and complex anatomy changes in patients. The imaging noise level from phantoms may also be different from that of real patients, which may impact the DIR validation results.

Additionally, some researchers use mathematical phantoms for validation.^3^, ^21^ This method capitalizes on known mathematical transformations, normally spline‐based deformation such as B‐splines^(21)^ or thin‐plate–splines,^(3)^ and applies a known deformation to a CT image to create an artificial deformed CT image. DIR algorithms are tested by registering these two images and comparing them with the known mathematical deformation. Again, it is difficult for mathematical phantoms to mimic the realistic anatomy changes in patients because simple spline‐based deformations could be very different from complicated patient anatomy changes during radiation treatment. Therefore, mathematical phantoms are inadequate for validating the DIR algorithms.

The purpose of this study is to create artificial CT images with known deformations that are able to simulate realistic patient anatomy changes so that these images can be used for volumetric validation of DIR algorithms at the voxel level. Although generating artificial images with known deformation is straightforward, it is not clinically relevant unless a few criteria are met. First, the artificial images must be created using population data to represent an entire population instead of a single patient. Second, the artificial images must simulate real clinical situations; therefore, the deformations cannot be arbitrarily generated and must be anatomically representative. Here we propose a novel approach to generating artificial images for DIR validation. First we used the population‐based modeling approach^22^, ^23^ to learn the actual anatomy changes associated with radiation treatment from cohorts of patients who received similar radiation treatments. The anatomy changes learned using this method were modeled to generate a random deformation between two CT images that would represent typical anatomy changes in a given cohort. This random deformation was used to generate pairs of artificial CT images with the appearance of real patient anatomy. The DIR algorithms were then applied to the artificial CT images and the registration results were compared against the known deformation to validate the DIR algorithms.

## II. MATERIALS AND METHODS

### A. Patient data

Twelve head and neck cancer patients who received IMRT at MD Anderson Cancer Center were retrospectively selected for this study and approved by the institutional review board of MD Anderson Cancer Center. These patients underwent daily photon irradiation for 32–35 fractions at 2 Gy per fraction. Each patient received a simulation CT scan for treatment planning and received daily CT scans using treatment room CT‐on‐rails (GE Healthcare, Milwaukee, WI) prior to each irradiation. As a part of our selection criteria, all CT scans should cover parotid glands and mandible. In addition, we did not include those patients having significant weight loss, tumor shrinkage or neck flexion, for which DIR could not be properly applied to the CT images to generate training DVFs.

### B. Learning anatomy variations

To ensure that the pairs of artificial images we created would show realistic anatomy changes, we first generated a known deformation between those images that would represent realistic anatomy changes during radiation therapy in a given cohort. This deformation could be learned from a given training dataset composed of patient images with anatomy changes. This learning process was derived from the active shape model proposed by Cootes et al.,^24^, ^25^ which can capture the most prominent shape variations of certain structures in a set of images through principal component analysis (PCA).^(26)^ This learning process later on will be applied to generating both the intrapatient variation model and the interpatient variation model. In our proposed method, we first selected a reference image from the training dataset and then performed DIR between the reference image and the other images. We used a dual‐force demons DIR algorithm,^(27)^ which was shown to have better accuracy and convergence than the original demons algorithm.^(28)^ The DVF resulting from this registration can be represented by

(1) $$d(\vec{x},t)=\{d_{x}(\vec{x},t),\;d_{y}(\vec{x},t),d_{z}(\vec{x},t)\}$$

where $d_{x}(\cdot ,t),\;d_{y}(\cdot ,t)$,and $d_{z}(\cdot ,t)$ represent the displacement field matrices from image t,t=1,2,…,N, to the reference image along the left–right (LR), anterior–posterior (AP), and superior–inferior (SI) directions, respectively, and $\vec{x}$ indexes the voxel location. The anatomy variations between the reference image and the other images are represented by these DVFs in a 3D space.

To create the active shape model, we let $\vec{d}(t)$ be the column‐wise vectorization of the DVF for image t, and let $D=\{\vec{d}(1),\;\vec{d}(2),\ldots ,\vec{d}(N)\}$ be the matrix consisting of N DVFs. First, we calculated the sample covariance matrix of D, denoted by Σ, as

(2) $$\sum =\frac{1}{N-1}\sum_{t=1}^{N}(\vec{d}(t)-\bar{d}){\left(\vec{d}(t)-\bar{d}\right)}^{T}$$

where *T* denotes the transpose, and the column vector $\bar{d}$ represents the mean deformation over *N* images along a specific direction and is calculated as

(3) $$\bar{d}=\frac{1}{N}\sum_{t=1}^{N}\vec{d}(t).$$

The representative anatomy variations in the training dataset can be obtained by calculating eigenvalues and eigenvectors of the covariance matrix Σ as

(4) $$\sum {\vec{\phi }}^{\left(j\right)}=\lambda^{\left(j\right)}{\vec{\phi }}^{\left(j\right)},j=1,2,\ldots ,N;$$

where $\lambda^{\left(j\right)}$ and ${\vec{\phi }}^{\left(j\right)}$ are the jth eigenvalue and eigenvector of *Σ*, respectively. Without loss of generality, we assumed $\lambda^{\left(1\right)}\geq \lambda^{\left(2\right)}\geq \ldots \lambda^{\left(N\right)}$, and that their corresponding eigenvectors represent different modes of variation. The PCA showed that a few of the eigenvectors corresponding to the large eigenvalues were able to capture most variations of the deformation. The principal modes, ${\vec{\phi }}^{\left(1\right)},{\vec{\phi }}^{\left(2\right)},\ldots ,{\vec{\phi }}^{\left(\hat{T}\right)}$, is a subset of $\left\{{\vec{\phi }}^{\left(j\right)}|j=1,2,\ldots ,N\right\}$ with $\hat{T}$ as the smallest number satisfying

(5) $$\sum_{j=1}^{\hat{T}}\lambda^{\left(j\right)}\geq \frac{\alpha }{100}\sum_{j=1}^{N}\lambda^{\left(j\right)},$$

where *α* is a value between 0 and 100. This equation acknowledges that the first $\hat{T}$ eigenvectors that represent the most prominent variations amount to at least α percent of the total variations that deviate from the mean deformation. Normally, α was set to a value between 80 and 95, and $\hat{T}«N$. The efficiency of the compact space representation can be evaluated by the variation space reduction, calculated as $(\hat{N‐T})/N$. In general, a larger α value was used if more variations existed in the model. The mean deformation d̅ and the principal modes of variation, ${\vec{\phi }}^{\left(1\right)},{\vec{\phi }}^{\left(2\right)},\ldots ,{\vec{\phi }}^{\left(\hat{T}\right)}$, composed the active shape model.

A new random deformation can be generated from the model using the following equation:

(6) $$d=\bar{d}+\sum_{j=1}^{\hat{T}}b_{j}{\vec{\phi }}^{\left(j\right)},$$

where $b_{j}$ is generated randomly and represents the deformation contributed by the jth mode of variation. To ensure that the deformation is reasonable and realistic, a maximum value, $D_{\text{max}}$, was enforced on the generation of random values ($b_{j}$) according to the following equation:

(7) $$\sum_{j=1}^{\hat{T}}\left(\frac{b_{j}^{2}}{\lambda^{\left(j\right)}}\right)\leq D_{\text{max}}^{2}$$

Essentially, a Gaussian distribution with zero mean and a diagonal covariance matrix composed of the eigenvalues $\lambda^{\left(j\right)}$ was assumed in generating the random values, and $D_{\text{max}}$ is the maximum Mahalanobis distance from the mean for the randomly generated parameters. $D_{\text{max}}$ was chosen to include a suitably large proportion of possible deformations. Using this method, we were able to generate random DVFs with realistic anatomy changes learned from the patient populations. These DVFs could be applied to the previously selected reference image to generate artificial CT images that resemble actual patient CT images.

### C. Creating artificial CT images

**Figure 1:**
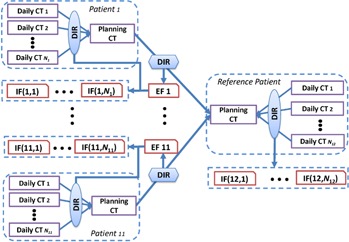
Illustration of the procedure to create the interpatient variation model and intrapatient variation model based on the training data from 12 head and neck cancer patients.

We applied the learning process described above to our dataset of head and neck cancer patients to generate pairs of artificial planning and daily CT images. The first step was to determine the interpatient anatomy variation in the population. We chose a patient who represented the approximate median of the population as the reference patient in terms of patients' weight and body mass index (BMI). This will facilitate the interpatient registration. As a preprocessing step, all planning CT and daily CT images were first aligned to the reference planning CT using cross‐correlation coefficient. Next, the largest dimension of a common space for these images was determined and all images were cropped to this size. The planning CT images of the remaining patients were registered to the planning CT image of the reference patient using the dual‐force demons DIR algorithm, creating 11 DVFs, EF 1, EF 2, …, EF 11 (Fig. 1). These DVFs composed the training dataset for the creation of the interpatient variation model. The parameter α was set to 90 in creating the interpatient variation model, meaning that the model represented at least 90% of the total variations in the entire cohort.

The next step was to determine the intrapatient variation over the course of treatment. For each patient, we registered the daily CT images to their respective planning CT image using the dual‐force demons DIR algorithm. The resulting DVFs characterize the intrapatient variations for each patient and reside in the planning CT space of each patient. Mathematically, let ${\vec{D}}_{i,j}^{\left(1\right)}$ denote the deformation vector at point $\vec{x}$ for patient i and fraction j. These DVFs should be further moved to a common space (i.e., the reference planning CT space). To do so, each intrapatient DVF was treated as three 3D images of magnitude of intrapatient variations in LR, AP, and SI directions and these images were deformed by the corresponding interpatient DVF (EF 1, EF 2, …, EF 11) to transfer them to the planning CT space of the reference patient, generating a set of DVFs, termed IF(1,1), IF$(1,1),\text{IF}(1,N_{1});\text{IF}(2,1),\ldots ,\text{IF}(2,N_{2}$); …; and IF$\left(1,1),\text{IF}(1,N_{1});\text{IF}(11,N_{11}\right)$, as shown in Fig. 1. Mathematically, ${\vec{f}}_{k}(\vec{x})$ denote the mapping defined by the interpatient DVF, EF k, and ${\vec{d}}_{i,j}^{\left(2\right)}(\vec{y})$ denote the DVF, IF(i,j). The aforementioned DVF mapping can be described as

(8) $${\vec{D}}_{i,j}^{\left(2\right)}(\vec{y})={\vec{D}}_{i,j}^{\left(1\right)}({\vec{f}}_{i}(\vec{x})).$$

We also registered the daily CT images to the planning CT image for the reference patient, producing DVFs named IF(12,1), …, IF(12, N_12_). These DVFs, denoted by IF(·,·), characterized the intrapatient variations of the population and composed the training dataset for the creation of the intrapatient variation model. The parameter α was set to 95 in creating the intrapatient variation model.

**Figure 2:**
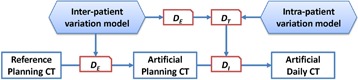
Illustration of the procedure to generate artificial planning CT and daily CT images using active shape models learned from the patient population.

The artificial CT images were generated using the variation models. We first used the interpatient variation model in Eq. (6) to generate a random DVF, $D_{E}$, and inverted it by using an iterative method^(29)^ for progressively refining the values of the inverse field. We then applied the inverse $D_{E}$ to the reference planning CT to generate an artificial planning CT image. Next, we used the intrapatient variation model in Eq. (6) to generate a random DVF, $D_{T}$, which was then deformed by $D_{E}$ to the space of the artificial planning CT image space resulting in a new DVF, $D_{I}$, and then we inverted $D_{I}$ and applied it to the artificial planning CT image to generate an artificial daily CT image (Fig. 2). Each pair of artificial planning CT and daily CT images represents the intrapatient anatomy variations during a treatment course. By varying the $D_{\text{max}}$ value in generating the daily CT images, we produced different degrees of anatomy changes that could happen between the planning CT and the daily CT.

Using this procedure, we generated two sets of artificial images, each set including one artificial planning CT image and three corresponding artificial daily CT images with varied deformation amounts. We used $D_{\text{max}}=3$ for the interpatient variation model to generate the planning CT image, and the $D_{\text{max}}$ was set to 2, 3.5, and 4.5 for the intrapatient variation model in generating the three daily CT images. We then used the dual‐force demons DIR algorithm to register each artificial daily CT image to the artificial planning CT image and generated a DVF, $D_{N}$, for each registration to be compared with the known deformation, $D_{I}$, which were randomly generated from the variation models. We subtracted $D_{N}$ from $D_{I}$ in the LR, AP, and SI directions and computed the total magnitude of difference at each voxel as the registration error. The registration errors at each voxel inside the mandible and each parotid gland were computed, and means and standard deviations (SDs) of these errors were calculated.

## III. RESULTS

### A. Dual‐force demons DIR algorithm

**Figure 3:**
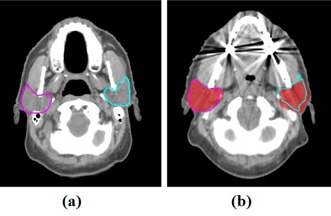
Parotid contours on the reference planning CT (a) was deformed to another patient using the dual‐force demons DIR algorithm. The deformed contours were compared with the manual contours (red colorwash) on this patient's planning CT (b).

The dual‐force demons DIR algorithm was used to generate the training DVFs. These training DVFs should be realistic for the latter anatomy variation learning; therefore, it might be necessary to evaluate the dual‐force demons DIR algorithm when applying to head and neck CT images. This registration algorithm directly calculates correspondences according to image intensity under the assumption that the intensity is consistent between two images. Because CT numbers are calibrated to the attenuation coefficient of water, this intensity‐based method is preferred for CT‐to‐CT registration. This algorithm was originally proposed by Wang et al.^3^, ^27^ and was validated for both intra‐ and interpatient registration for head and neck cancer radiotherapy with reasonably good results.^12^, ^30^, ^31^ Here we also provided a quantitative and qualitative evaluation of this algorithm when applying to generate interpatient DVFs. The clinical manual contours of left and right parotid glands were used for the evaluation. The contours on the reference planning CT were deformed to the other 11 planning CT spaces using the interpatient DVFs (EF 1, EF 2, …, EF 11). The deformed contours were compared with the manual contours using the Dice similarity coefficient (DSC) and the mean surface distance (MSD), as shown in Table 1. In addition, we illustrated the comparison of deformed contours and manual contours for one patient in Fig. 3. These results showed that the interpatient registration was reasonably good mostly by considering the interobserver variability in drawing the contours on different patients. However, we acknowledge the existence of registration error in some spatial locations. We also found that the dental artifacts did not show significant impact on the DIR algorithm, which was consistent with previous findings.^30^, ^31^ This effect is possibly owing to the regularization on the DVF in the DIR algorithm.^(3)^ The DVFs generated from the DIR algorithm is reasonable in most cases with the present of dental artifacts, therefore minimizing the effect on the PCA results in the learning process.

**Table 1:** The parotid contours on the reference planning CT were deformed to other 11 patients and compared with the manual contours using Dice similarity coefficient (DSC) and mean surface distance (MSD).

	*Right Parotid*		*Left Parotid*	
*Patient No*	*DSC (%)*	*MSD (mm)*	*DSC (%)*	*MSD (mm)*
1	74.3	3.3	74.6	3.1
2	72.7	3.0	79.2	2.4
3	63.9	3.8	70.1	3.1
4	59.7	5.1	70.7	4.0
5	53.6	6.1	58.4	5.3
6	84.6	1.8	82.5	2.2
7	69.1	3.9	80.4	3.2
8	33.5	2.9	63.2	3.4
9	51.3	5.4	71.7	3.7
10	64.8	3.7	82.2	2.1
11	82.5	2.2	86.0	2.0
Mean	64.6	3.7	74.5	3.1
SD	14.8	1.3	8.6	1.0

SD=standard deviation.

### B. Creating artificial CT images

In the creation of the interpatient variation model, a number of 5 principal modes out of a total of 11 modes were enough to represent 90% of interpatient variations between the 12 patients, and the variation space was reduced by 55%. In the creation of the intrapatient variation model, 396 deformations were used in the training process, and a number of 12 principal modes out of a total of 396 modes were enough to represent 95% of the intrapatient variations, achieving a variation space reduction of 97%. The interpatient variation model did not show efficient compact space representation because of a small number of training datasets (11 interpatient DVFs). A variation space reduction of 97% for intrapatient variation model showed that the intrapatient variations were consistent in this population, and a few principal modes of variation were able to represent most intrapatient anatomy changes in a head and neck radiation treatment course.

**Figure 4:**
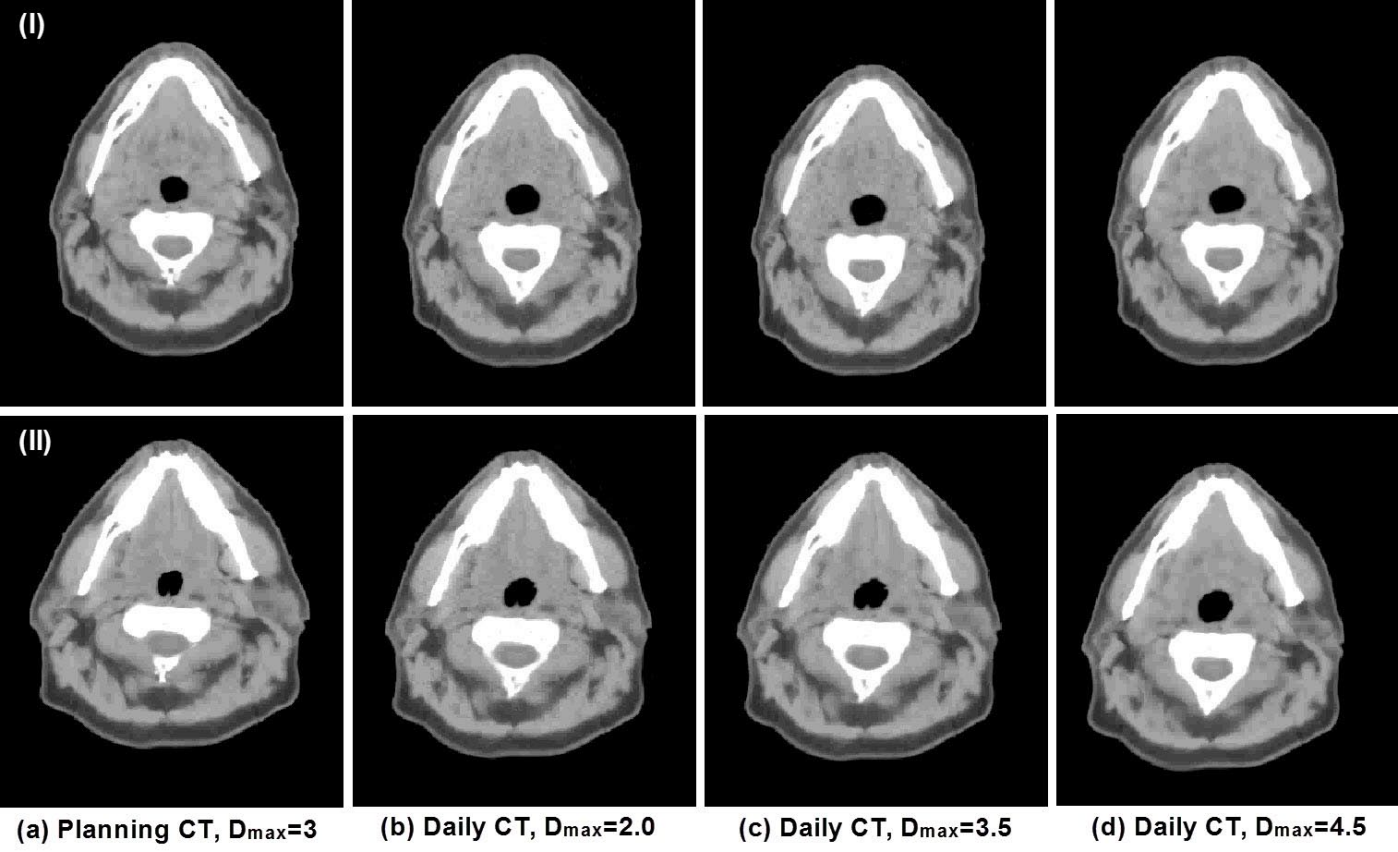
Two sets of artificial CT images generated from active shape models with each set in a row. The first column is the artificial planning CT, and the other three columns are artificial daily CT images with varied levels of variations specified by $D_{\text{max}}$.

**Figure 5:**
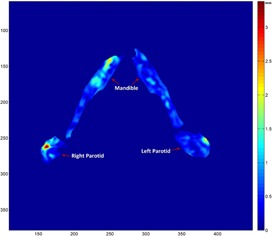
Illustration of the spatial distribution of registration errors at each voxel inside the parotids and mandible on an axial slice. The registration error was computed by comparing the calculated deformation with the known deformation generated randomly from the variation models. The registration error is small in most areas inside both structures.

Two sets of artificial CT images, representing two different simulated patients, are shown in Fig. 4, each set containing one planning CT image ($D_{\text{max}}=3$) and three corresponding daily CT images with varied deformation amounts ($D_{\text{max}}$ set to 2, 3.5, and 4.5). We observed a difference between the planning CT images in the two sets and subtle anatomy changes from the planning CT to the daily CT images within each set. These images realistically simulated the clinical ones and were useful for the validation of DIR in a clinical environment. The registration errors in the mandible and parotid glands between the planning CT and daily CT images are quantified in Table 2, compared against the mean deformations for each organ between the artificial planning CT and daily CT images in Table 3. Note that $D_{\text{max}}$ stipulates the maximum Mahalanobis distance from the mean in generating the random values (bj) in Eq. (7). Smaller random deformation could be generated with a larger $D_{\text{max}}$. Therefore, it is not a surprise that the mean deformation in simulation set 1 is smaller for $D_{\text{max}}=4.5$ than that for $D_{\text{max}}=3.5$ in Table 3. The results illustrated the feasibility of using known deformation for quantifying deformable registration errors. In addition, the registration errors at each voxel inside the parotids and mandible, exemplified in Fig. 5 with the registration error color‐coded, show that registration error ranged from 0 mm to 3.3 mm in this illustration, with the largest error in the lateral right parotid region.

**Table 2:** Quantitative registration evaluation for the mandible and the parotid glands between the artificial planning CT and daily CT images shown in Fig. 4. The registration error was evaluated by comparing the calculated deformation against the known deformation generated randomly from the variation models.

*Simulation Set*	*Organ*	$Daily\;CT\;D_{\text{max}}=2.0\;Mean\pm SD\;(mm)$	$Daily\;CT\;D_{\text{max}}=3.5\;Mean\pm SD\;(mm)$	$Daily\;CT\;D_{\text{max}}=4.5\;Mean\pm SD\;(mm)$
	Left parotid	0.74±0.49	1.06±0.62	0.71±0.46
1	Right parotid	0.69±0.51	1.05±0.81	0.75±0.55
	Mandible	0.68±0.38	0.96±0.54	0.96±0.58
	Left parotid	0.69±0.79	0.78±0.67	0.83±0.68
2	Right parotid	0.60±0.48	0.73±0.58	0.93±0.64
	Mandible	0.71±0.39	0.85±0.52	0.98±0.61

**Table 3:** The mean magnitude of deformation for organs of interest between the artificial planning CT and daily images shown in Fig. 4. They were computed from the known deformation generated randomly from the variation models.

*Simulation Set*	*Organ*	$Daily\;CT\;D_{\text{max}}=2.0\;Mean\pm SD\;(mm)$	$Daily\;CT\;D_{\text{max}}=3.5\;Mean\pm SD\;(mm)$	$Daily\;CT\;D_{\text{max}}=4.5\;Mean\pm SD\;(mm)$
	Left parotid	9.15±0.71	17.40±0.94	9.26±0.99
1	Right parotid	7.86±0.70	14.60±0.88	7.73±0.86
	Mandible	7.66±0.68	14.70±1.22	8.65±0.76
2	Left parotid	8.64±0.69	10.70±0.89	19.55±1.00
	Right parotid	7.13±0.68	8.13±0.83	16.74±0.91

## IV. DISCUSSION

We proposed a method to generate artificial CT images for DIR validation by learning the anatomy changes associated with radiation treatment from patient populations. This method captured the most prominent anatomy variations in a population and generated a compact representation of the variations using an active shape model. Artificial CT images generated with this method could potentially simulate patient anatomy changes during radiation treatment; therefore, using these artificial CT images for validation is clinically feasible. Because this process is purely mathematical, there is no need to build a physical phantom, which makes DIR validation simple and usable in many clinics with their own patient data. We demonstrated the efficacy of the proposed method by generating artificial planning CT and daily CT images in an IMRT course for head and neck cancer that have complicated anatomy changes during radiation treatment and may pose challenges for deformable registration. However, testing our method on the head and neck site with success provided us with confidence that this method can be used on other sites as well.

**Figure 6:**
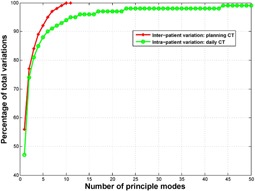
Percentage of total variations represented as a function of the number of principal modes needed in creating the active shape model. The total number of principle modes for interpatient variation and intrapatient variation is 11 and 396, respectively.

The active shape models have been widely used in varied image‐processing applications such as model‐based image registration and segmentation.^32^, ^33^, ^34^, ^35^ A similar technique using PCA to capture prominent variations has also been used to create respiratory models from 4D CT images.^36^, ^37^, ^38^ An important characteristic of this technique is the compact representation of variations learned from the population. This method can disregard the redundant information in the population data and keep the most essential representation of the variations, thus enabling the generation of artificial images with the most common variations that one can observe. We investigated the efficiency of this compact representation method. For the two models of variation that were created in this study, we plotted the percentage of total variations captured by the model versus the number of principal modes needed (Fig. 6). We found that five principal modes were able to represent 90% of the total variations for interpatient variation and six principal modes were able to represent 90% of the total variations for intrapatient variation models. However, due to a different number of total modes (11 for interpatient and 396 for intrapatient variation models), intrapatient variation model showed much higher efficiency in variation space reduction. Also, because of a small number of total modes for interpatient variation model, the interpatient variation reached 100% much faster than the intrapatient variation in Fig. 6.

**Figure 7:**
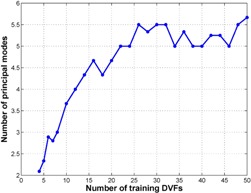
The number of principal modes needed to achieve 90% of the total variations as a function of the number of training deformation vector fields (DVFs). The number of principal modes was obtained through interpolation. For example, with 20 training DVFs, four and five principal modes achieved 88% and 91% of the total variations, respectively. After interpolation, 4.7 principal modes were needed to achieve 90% of the total variations.

Learning based on the PCA modeling is limited by the training data. The artificial images can represent only the variations learned from the training DVFs. To provide a basis for realistic simulations of clinical CT images and optimally capture the variation that can be encountered in the clinic, the training DVFs need to contain as much of that variation as possible. However, using more training DVFs is not necessarily better for this approach because of limitations in computational resources. In addition, the training DVFs contain a lot of redundancy, so the variations could be represented by a small number of principal modes. Therefore, it would be interesting to investigate what amount of training DVFs is necessary to build an acceptable model. To do so, we studied the number of modes needed to represent 90% of the total variations in the training DVFs as a function of the amount of training DVFs, using training DVFs that were used to create the intrapatient variation model. As shown in Fig. 7, when the training DVFs were not enough, the number of modes needed to achieve 90% of the total variations increased as the number of training DVFs increased. But at a certain stage, adding more training DVFs did not contribute to new representative variations and the number of modes needed stayed approximately the same, indicating that the training DVFs were enough to represent most variations at that stage. In this example, a minimum of about 26 training DVFs were needed for the active shape model to capture more than 90% of the total variations in the population.

This study is limited by the registration errors of the DIR algorithm used to create the training DVFs, including the interpatient registration error and the intrapatient registration error. The interpatient registration error is the major source of uncertainty for this study. The results shown in the Results section A indicated that large registration error might exist at some locations and this error could propagate into the variation models. Interpatient registration error has effects on both the interpatient and intrapatient variation models. It affects the interpatient variation model directly, but the effect could be mitigated by PCA unless strong pattern of errors existed in most patients used for the training. Interpatient registration error affects the intrapatient variation model indirectly when it was used to relocate the intrapatient DVFs through Eq. (8). This effect is possibly smeared out by a large amount of intrapatient DVF samples and relatively small intrapatient deformation. Intrapatient registration error, in general, is small and affects intrapatient variation model only. We did not include those patients having significant anatomy changes that prevent DIR from working properly, while normal tumor shrinkage or weight loss cases were included. This further reduced potential intrapatient registration error. On the other hand, models built from this training dataset may not be able to simulate those real clinical situations with large anatomical changes. A potential solution is to construct a tumor shrinkage model^(39)^ and simulate the changes in artificial CT images, which will be a subject of our future research.

This study focused on simulating intrapatient anatomy changes during radiation therapy. Although both interpatient and intrapatient variation models were created to generate the artificial CT images, we used the artificial CT images to validate the DIR algorithms for intrapatient registration only. The interpatient variation model is simply used to generate a random new patient that is not the same as the reference patient. The variation in this model is limited and may not be realistic because interpatient variation is more susceptible to registration errors due to large interpatient anatomical variations. The interpatient variation model needs further investigation because the interpatient variations are much more complicated than the intrapatient variations. More training datasets are required to account for an acceptable percentage of interpatient variations.

Another limitation of this study is that we used only one DIR algorithm to create the inter‐ and intrapatient variation models for artificial CT image generation. This could possibly create a bias if we attempted to validate the DIR algorithm used to create the models. The inherent patterns or regularization in the DIR algorithm might be incorporated in the variation models, which favors the evaluation of this DIR algorithm. To overcome this limitation, one may use different DIR algorithms to create the variation models, which will add variations to algorithm specific patterns or regularization in the models, thus potentially removing the bias. In addition, this approach is also limited by the accuracy of training DVFs, the ability of these DVFs to represent high spatial frequency deformations, and the failure of DIR algorithm to produce realistic tissue deformation in featureless subvolumes. For example, the regularization in DIR algorithm tends to smoothing DVFs, thus possibly reducing the capability of representing the complexity and discontinuities of the deformation in some real patients.

The anatomy changes might be correlated to the time point during a treatment course,^(40)^ which suggests the possibility to construct a model as a function of time so that the artificial CT image can be simulated for a specific stage in the treatment course. This will be a subject of our future study. Our current approach using active shape models took into account anatomy shape changes only. However, the same anatomy may appear differently on CT images under different scanning conditions. These tissue appearance changes also may be learned from patient populations by taking advantage of active appearance models.^(41)^ On the other hand, using an appearance model may reduce the impact from noise and artifacts. Our current approach is limited by transferring noise and artifacts from template image, thus leaving a signature of the underlying deformation, which may potentially skew the DIR accuracy. In addition, in actual clinical situations, some structures may contain objects that appear and disappear in different scans — for example, air pockets in the rectum or esophagus. Such objects will create a non‐correspondence issue and affect the DIR. Therefore, artificial images simulating this situation must be created to thoroughly validate DIR algorithms. A previous study^(42)^ has demonstrated the possibility of adding a simulated tumor or other objects to an existing image. In our future research, we will include the active appearance model and the noncorresponding objects to artificial CT images to generate more realistic artificial images for the validation of DIR algorithms.

## V. CONCLUSIONS

We proposed a method to learn the anatomy changes during radiation therapy in patient populations and created active shape models for the purpose of generating artificial CT images to validate DIR algorithms. Artificial CT images generated from this method potentially simulated the actual patient anatomy changes during radiation treatment. We demonstrated the practicability of the proposed method in simulating anatomy changes during IMRT for patients with head and neck cancer.

## ACKNOWLEDGMENTS

This work was supported in part by the National Institutes of Health through Cancer Center Support Grant CA016672 and a sponsored research grant from Varian Medical Systems. This work was also performed in partial fulfillment of the requirements for the Ph.D. degree from The University of Texas Graduate School of Biomedical Sciences at Houston. The authors would like to thank Sarah Bronson from the Department of Scientific Publication at MD Anderson Cancer Center for reviewing the manuscript.
